# Galactorrhea as a side effect of antidepressant drugs. A case report

**DOI:** 10.1192/j.eurpsy.2021.2052

**Published:** 2021-08-13

**Authors:** A.I. Willems Aguado, L. Garcia, C. Rodriguez

**Affiliations:** 1 Salud Mental, CSM Cangas del Narcea, Cangas del Narcea, Spain; 2 Csm Eria, SESPA, Oviedo, Spain; 3 Csm La Calzada, SESPA, Gijon, Spain

**Keywords:** galactorrhea, side effects, case report, antidepressant drugs

## Abstract

**Introduction:**

Galactorrhea wiht antidepressants SSRIs or SNRI is a rarely adverse effect. Some authors believe that the risk of galactorrhea in women who use SSRIs is 8 times higher than in patients treated with other types of drugs. Serotonin is believed to be a potent physiological stimulator of prolactin release.Prolactin stimulates the growth of the mammary glands and the galactorrhea. The SSRIs would activate the serotonergic pathways, these in turn would stimulate the release of prolactin directly in the pituitary and in the hypothalamus, inhibiting the release of dopamine and increasing the release of stimulating factors. The main inhibitor of prolactin secretion is dopamine.

**Objectives:**

The objective is to reveal this rare complication through the report of a clinical case

**Methods:**

A 45-year-old woman with a diagnosis of mixed anxiety-depressive disorder. Treatment with 20 mg of escitalopram was started, with a good therapeutic response, but with breast pain and swelling. She was switched to duloxetine 60 mg, with a good response and adequate tolerance. At 6 months of treatment, she begins to present breast pain and yellow-green breast discharge, with elevated prolactin levels and normal cranial MRI.

**Results:**

She was diagnosed with functional hyperprolactinemia, and treatment with vortioxetine was started. Finally, the Prolactin levels normalize.

**Conclusions:**

Galactorrhea is a very rare and annoying side effect that can lead to discontinuation of treatment and requires a change in the therapeutic strategy.

**Disclosure:**

No significant relationships.

